# Screening Frailty Among Community-Dwelling Older Adults: A Cross-Sectional Study Comparing the Edmonton Frail Scale and the Clinical-Functional Vulnerability Index-20

**DOI:** 10.3390/geriatrics11050141

**Published:** 2026-10-07

**Authors:** Ana Paula Felix Arantes, Fabiana Machado Pires, Rodrigo Franco de Oliveira, Maria Fernanda Pereira Costa Carvalho, Lorenna Guimarães Pereira, Vitor Oliveira Arantes, Mariana de Jesus Ferreira, Guilherme Martins Oliveira, Maria Fernanda Gomes Pereira, Gabriela Evangelista Nascimento, Renato Canevari Dutra da Silva, Adriana Vieira Macedo Brugnoli, Caroline Brand, Deise Aparecida de Almeida Pires Oliveira

**Affiliations:** 1 Department of Physical Therapy, Universidade de Rio Verde, Fazenda Fontes do Saber, Campus Universitário, P.O. Box 104, Rio Verde 75901-970, GO, Brazil; fabianamachado@unirv.edu.br (F.M.P.); marianadejesusrv9233@gmail.com (M.d.J.F.); guilherme27112005@gmail.com (G.M.O.); renatocanevari@unirv.edu.br (R.C.D.d.S.); adriana.brugnoli@unirv.edu.br (A.V.M.B.); 2 Postgraduate Program in Human Movement and Rehabilitation, Evangelical University of Goiás, Campus Anápolis, Av. Universitária, Km 3.5, Cidade Universitária, Anápolis 75083-515, GO, Brazil; rodrigofranco65@gmail.com (R.F.d.O.); mafeapccarvalho0301@gmail.com (M.F.P.C.C.); deisepyres@gmail.com (D.A.d.A.P.O.); 3 Department of Medicine, Universidade de Rio Verde, Fazenda Fontes do Saber, Campus Universitário, P.O. Box 104, Rio Verde 75901-970, GO, Brazil; lorennaguimaraes16@gmail.com (L.G.P.); vitor230417@gmail.com (V.O.A.); gomesmariafernanda41@gmail.com (M.F.G.P.); gabievangelista.n@gmail.com (G.E.N.); 4 IRyS Research Group, School of Physical Education, Pontificia Universidad Católica de Valparaíso, Av. Universidad 651, Curauma, Valparaíso 2581967, Chile; caroline.brand@pucv.cl

**Keywords:** frailty, aged, mass screening, Edmonton Frail Scale, geriatric assessment

## Abstract

**Background/Objectives:** The Edmonton Frail Scale (EFS) and the Clinical-Functional Vulnerability Index-20 (IVCF-20) are multidimensional screening instruments that differ in the domains assessed, scoring systems, and classification thresholds, which may lead to different frailty-related classifications. Although both instruments are used to identify vulnerability in older adults, the extent to which their classifications agree remains insufficiently characterized. This study compared EFS and IVCF-20 classifications among community-dwelling older adults by examining category distribution, cross-classification, agreement, and correlation between total scores. **Methods:** This cross-sectional observational study included 110 community-dwelling adults aged ≥60 years recruited by convenience sampling from a public community center in Rio Verde, Goiás, Brazil. Participants completed the EFS and IVCF-20 during the same assessment session. Agreement across the original three ordinal categories was evaluated using linear-weighted Cohen’s kappa, and the relationship between total scores was assessed using Spearman’s rank correlation coefficient. **Results:** The EFS classified 43.6% of participants as non-frail, 31.8% as pre-frail, and 24.5% as frail, whereas the IVCF-20 classified 43.6% as robust, 37.3% as potentially frail, and 19.1% as frail. Exact agreement was observed in 65.5% of participants (95% CI: 56.2–73.7%), and linear-weighted Cohen’s kappa indicated moderate agreement (κ = 0.543; 95% CI: 0.415–0.670; *p* < 0.001). Most discordant classifications occurred between adjacent categories. Total scores were strongly positively correlated (Spearman’s ρ = 0.745; *p* < 0.001). **Conclusions:** The EFS and IVCF-20 showed a strong correlation between total scores but only moderate agreement between frailty-related classifications. Although the instruments capture related dimensions of vulnerability, their classifications should not be considered interchangeable.

## 1. Introduction

Population aging has increased the need for healthcare strategies capable of identifying older adults at risk of functional decline before substantial loss of independence occurs. Within this context, frailty has become an important clinical and public health construct because it reflects decreased physiological reserve and increased vulnerability to stressors and is associated with adverse outcomes such as falls, disability, hospitalization, and mortality [1,2,3]. Contemporary approaches to healthy aging have consequently emphasized person-centered assessment of physical and mental capacities and functional ability, particularly in primary and community care settings [4]. Early recognition of frailty and related vulnerabilities may therefore support timely multidimensional interventions and more appropriate allocation of healthcare resources [4,5].

Frailty is nevertheless a heterogeneous construct for which no single operational definition or assessment approach has been universally adopted. Two influential conceptual traditions illustrate this heterogeneity. The frailty phenotype proposed by Fried et al. conceptualizes frailty primarily through physical manifestations, including unintentional weight loss, exhaustion, weakness, slow walking speed, and low physical activity [2]. In contrast, the deficit-accumulation approach developed by Mitnitski and Rockwood conceptualizes frailty as the cumulative burden of health deficits spanning multiple systems and dimensions [6]. These conceptual differences have contributed to the development of numerous assessment instruments that vary in the domains evaluated, scoring and classification approaches, feasibility, and intended setting. Reviews of frailty assessment instruments have demonstrated substantial heterogeneity in their structure and dimensional coverage, highlighting the importance of considering the construct assessed and the context of application when selecting and interpreting frailty measures [7,8].

Among multidimensional instruments, the Edmonton Frail Scale (EFS) provides a relatively brief assessment incorporating cognition, general health status, functional independence, social support, medication use, nutrition, mood, continence, and functional performance. Originally developed by Rolfson et al., the EFS was designed to permit frailty assessment without requiring specialist geriatric expertise [9]. Its measurement properties have subsequently been investigated across different populations and settings, and a recent systematic review and meta-analysis found overall evidence supporting the reliability and construct validity of the original EFS, particularly in stable clinical and community populations [10].

The Clinical-Functional Vulnerability Index-20 (IVCF-20) was developed and validated in Brazil as a rapid multidimensional screening instrument for identifying clinical-functional vulnerability in older adults. It comprises 20 items covering age, self-perceived health, activities of daily living, cognition, mood, mobility, communication, and multiple comorbidities [11]. Importantly, the IVCF-20 is a screening instrument rather than a diagnostic reference standard; its purpose is to facilitate rapid recognition of older adults who may require further assessment and care planning [11]. Thus, although the EFS and IVCF-20 overlap in several domains relevant to frailty and functional vulnerability, their differing structures and intended constructs mean that interchangeability should not be assumed. Differences in their domains, weighting, scoring systems, and classification thresholds may result in the same individual being assigned to different frailty-related categories.

This distinction is particularly relevant in community-dwelling populations, where multidimensional frailty is common but prevalence estimates vary considerably according to the population and assessment approach. A 2024 systematic review and meta-analysis involving 40,597 community-dwelling older adults reported substantial heterogeneity in multidimensional frailty estimates and identified differences according to characteristics such as age, sex, education, and marital status [12]. More recent evidence also indicates that multidimensional frailty is associated with a broad range of demographic, psychosocial, and health-related factors, including lower education, depressive symptoms, poor self-rated health, chronic disease burden, and fear of falling [13]. These findings provide a rationale for exploring whether patterns of association with participant characteristics differ according to the screening instrument used.

Direct comparison of the EFS and IVCF-20 is clinically relevant, but evidence regarding their concordance in community-dwelling older adults remains limited. Carneiro et al. directly compared the two instruments in a Brazilian community-dwelling population and reported moderate agreement between their classifications and a strong positive correlation between total scores [14]. However, correlation between total scores does not necessarily imply agreement in categorical classification, and an overall agreement coefficient alone does not indicate where classification discrepancies occur. Examining the cross-classification of frailty-related categories may therefore provide additional information on whether discordance occurs predominantly between adjacent categories or reflects more substantial differences in classification. Moreover, because the EFS and IVCF-20 assess overlapping but non-identical domains, examining sociodemographic and health-related characteristics associated with each classification may help contextualize differences between the instruments, provided that these associations are interpreted as exploratory and not as evidence of independent predictors or causal relationships.

Accordingly, the primary aim of this study was to compare frailty-related classifications obtained with the EFS and IVCF-20 among community-dwelling older adults by examining the distribution and cross-classification of categories, agreement between classifications, and correlation between total scores. As a secondary exploratory aim, we examined sociodemographic and health-related characteristics associated with frailty classification according to each instrument. Neither instrument was treated as a diagnostic reference standard; rather, the analyses were designed to characterize their concordance and differences in classification within the study population.

## 2. Materials and Methods

### 2.1. Study Design and Sample

This cross-sectional observational study was conducted at the CONVIVER Community Center, a municipal community facility for older adults located in Rio Verde, Goiás, Brazil, between March and June 2024. Participants were recruited through convenience sampling among individuals attending activities at the center during the study period. This study was reported in accordance with the Strengthening the Reporting of Observational Studies in Epidemiology (STROBE) guidelines.

The inclusion criteria were (1) age ≥60 years; (2) ability to communicate and understand the assessment procedures; and (3) absence of significant cognitive impairment, operationally defined in the study protocol as a Mini-Mental State Examination (MMSE) score ≥19 [15,16]. The exclusion criteria were (1) clinical conditions that precluded participation in the functional assessments; (2) acute illness at the time of data collection; and (3) incomplete assessment with either the Edmonton Frail Scale (EFS) or the Clinical-Functional Vulnerability Index-20 (IVCF-20). Individuals who declined to participate were not enrolled in the study.

At the time of data collection, 125 individuals attending activities at the community center were considered for participation. Eight did not proceed to the complete study assessment: four were younger than 60 years, two declined to participate, one had an MMSE score <19, and one had a clinical condition that precluded participation in the functional assessment (wheelchair use). Thus, 117 participants proceeded to the study assessments. Seven participants had incomplete data for at least one of the two frailty-related instruments (EFS or IVCF-20) and were therefore excluded from the comparative analyses. The final analytical sample therefore comprised 110 participants with complete data for both instruments, all of whom were included in the analyses.

No formal a priori sample-size calculation was performed because recruitment was based on a convenience sample of individuals attending the community center during the predefined study period. The statistical precision and adequacy of the final sample for the primary agreement analysis were evaluated considering the final sample size and are described in Section 2.4.

### 2.2. Procedures

Participants were invited to take part in the study through face-to-face contact during breaks in routine activities at the CONVIVER Community Center for Older Adults. During the initial approach, potential participants received information about the study objectives, assessment procedures, voluntary nature of participation, anonymity, and confidentiality. Individuals who agreed to participate provided written informed consent before any study procedures were initiated. Formal authorization to conduct the study at the CONVIVER Community Center was obtained from the institution’s coordinators before the beginning of data collection.

Data collection was conducted individually and face-to-face at the community center by researchers who had undergone prior training and standardization of the study procedures. Before data collection began, members of the research team were trained in the administration of the standardized data collection form and assessment procedures, after which the researchers responsible for data collection were selected. Sociodemographic and clinical information was obtained through direct interviews and, when available, consultation of participants’ registration records.

All assessments were completed during a single session in a quiet and private environment at the community center. The instruments were administered according to the predefined sequence established in the data collection form, with the EFS administered before the IVCF-20. Both instruments were administered to each participant by the same researcher. Formal blinding between instruments was therefore not possible; however, total scores and corresponding classifications were not calculated during the assessment session. Scoring and classification of the instruments were performed only after completion of data collection. Thus, the researcher administering the IVCF-20 was not aware of the participant’s final EFS score or frailty classification at the time of administration.

The assessment session lasted approximately 20–50 min, depending on the amount of information required and the participant’s individual pace and comprehension. Assessments could be interrupted at any time at the participant’s request or if a clinical condition arose that prevented safe continuation of the procedures.

### 2.3. Instruments

The primary variables of interest were the classifications and total scores obtained with the Edmonton Frail Scale (EFS) and the Clinical-Functional Vulnerability Index-20 (IVCF-20) [9,11]. The classifications were analyzed according to the established cut-off points for each instrument, whereas total scores were used to examine the relationship between the two measures. Secondary variables included sociodemographic and health-related characteristics.

Sociodemographic variables included sex (female or male), self-reported race/skin color (White, Brown, or Black), age group (60–74, 75–84, or ≥85 years), marital status (with or without a partner), number of children (0, 1–2, 3–5, or >5), educational level (<1, 1–4, 5–8, or ≥9 years), family income (≤1, >1–3, or >3 minimum wages), and retirement status (yes or no). Health-related variables included self-perceived health (excellent, good, regular, poor, or very poor), smoking status (never, former, or current smoker), alcohol consumption (never, former, or current), depressive symptoms, muscle mass status (preserved or reduced), risk of falls (low, moderate, or high), and fear of falling (low, moderate, or high).

Cognitive function was assessed using the Mini-Mental State Examination (MMSE) as part of the eligibility assessment [15,16]. As described in Section 2.1, an MMSE score ≥ 19 was operationally established in the study protocol as the criterion for inclusion. The MMSE was therefore used to determine eligibility and was not subsequently analyzed as an independent health-related variable associated with the EFS or IVCF-20 classifications.

Depressive symptoms were assessed using the 15-item Geriatric Depression Scale (GDS-15), which consists of 15 dichotomous items answered as “yes” or “no”, with each item scored as 0 or 1 according to the direction of the question [17,18]. Total scores range from 0 to 15, with higher scores indicating a greater burden of depressive symptoms. In the present study, depressive symptoms were considered present when the total GDS-15 score was ≥6 [18].

Functional mobility and risk of falls were assessed using the Timed Up and Go Test (TUG), originally described by Podsiadlo and Richardson [19]. Participants were instructed to rise from a chair, walk three meters at a comfortable and safe pace, turn, return to the chair, and sit down, while the time required to complete the task was recorded in seconds. Longer completion times indicate poorer functional mobility and greater risk of falls. For categorical analysis, participants were classified as having low (<10 s), moderate (10–20 s), or high (>20 s) risk of falls, according to previously reported criteria [20].

Fear of falling was assessed using the Falls Efficacy Scale–International (FES-I), a 16-item instrument that evaluates concern about falling during a range of daily activities [21]. Each item is rated on a four-point scale, resulting in a total score ranging from 16 to 64 points, with higher scores indicating greater concern about falling. For categorical analyses, participants were classified as having low (16–19 points), moderate (20–27 points), or high (28–64 points) fear of falling, according to previously established cut-off points [21].

Muscle mass status was assessed using calf circumference as an anthropometric proxy. Participants were evaluated in the standing position with their feet approximately 20 cm apart. A nonelastic measuring tape was positioned perpendicular to the longitudinal axis of the lower leg, and the measurement was obtained at the point of greatest calf circumference. Muscle mass was classified as reduced when calf circumference was <34 cm in men and <33 cm in women; values equal to or above these thresholds were classified as preserved [22].

Frailty was assessed using the EFS, a multidimensional instrument originally developed by Rolfson et al. [9] and subsequently culturally adapted and validated for the Brazilian population [23]. The EFS comprises nine domains: cognition, general health status, functional independence, social support, medication use, nutrition, mood, continence, and functional performance. It combines self-reported information with performance-based assessment and yields a total score ranging from 0 to 17 points, with higher scores indicating greater frailty. In the present study, participants were classified as non-frail (0–4 points), pre-frail (5–6 points), or frail (≥7 points).

The IVCF-20 was developed and validated in Brazil by Moraes et al. as a rapid multidimensional screening instrument for clinical-functional vulnerability in older adults [11]. It comprises 20 items distributed across eight domains: age, self-perceived health, activities of daily living, cognition, mood, mobility, communication, and multiple comorbidities. The total score ranges from 0 to 40 points, with higher scores indicating greater clinical-functional vulnerability. According to the established cut-off points, participants were classified as robust (0–6 points), potentially frail (7–14 points), or frail (≥15 points). In the present study, the IVCF-20 was used as a comparator screening instrument rather than as a diagnostic reference standard for the EFS.

### 2.4. Statistical Analysis

Data were analyzed using Python (version 3.13.5), with the pandas (version 2.2.3), SciPy (version 1.17.0), and statsmodels (version 0.14.6) libraries [24,25,26,27]. All statistical tests were two-sided, and statistical significance was set at *p* < 0.05.

Descriptive statistics were used to characterize the study population. Categorical variables are presented as absolute and relative frequencies, whereas continuous variables are summarized using appropriate measures of central tendency and dispersion according to their distributions. Frailty-related classifications were defined according to the established cut-off points for the EFS and IVCF-20 described in Section 2.3.

For exploratory analyses of sociodemographic and health-related characteristics associated with frailty, the classifications of each instrument were dichotomized to reduce sparse cell counts. For the EFS, participants classified as non-frail or pre-frail (scores 0–6) were grouped as non-frail and compared with participants classified as frail (scores ≥ 7). For the IVCF-20, participants classified as robust or potentially frail (scores 0–14) were grouped as non-frail and compared with those classified as frail (scores ≥ 15).

Associations were examined using Pearson’s chi-square test when its assumptions were satisfied. Yates’ continuity correction was applied to 2 × 2 contingency tables with adequate expected cell frequencies. Fisher’s exact test was used for sparse 2 × 2 contingency tables, whereas Monte Carlo simulation was used to estimate *p*-values for larger contingency tables with insufficient expected cell frequencies, using 200,000 resamples and a fixed random seed (20260905) to ensure reproducibility. Because these analyses involved multiple sociodemographic and health-related characteristics and were exploratory in nature, no adjustment for multiple comparisons was applied; consequently, the corresponding *p*-values should be interpreted cautiously.

Agreement between the EFS and IVCF-20 was evaluated using their original three ordinal classification categories. A 3 × 3 cross-classification table was constructed, and exact agreement was calculated as the proportion of participants assigned to corresponding categories by both instruments, with its 95% confidence interval. Because the categories are ordinal, linear-weighted Cohen’s kappa was used as the primary agreement coefficient and is reported with its 95% confidence interval. Unweighted Cohen’s kappa was additionally calculated as a secondary measure of agreement [28,29].

The relationship between EFS and IVCF-20 total scores was assessed using Spearman’s rank correlation coefficient (ρ). The choice of Spearman’s correlation was based on assessment of the score distributions using the Shapiro–Wilk test, which indicated significant departures from normality. Pearson’s correlation coefficient was additionally calculated as a sensitivity analysis to evaluate the consistency of the association.

The IVCF-20 was treated as a comparator screening instrument rather than as a diagnostic reference standard. Accordingly, the primary comparative analyses focused on agreement between classifications and correlation between total scores. Measures requiring designation of one instrument as a diagnostic reference standard, including sensitivity, specificity, predictive values, likelihood ratios, and receiver operating characteristic (ROC) curve analysis, were not included in the revised analyses.

No formal a priori sample-size calculation was performed because participants were recruited through convenience sampling during the predefined study period. Sample-size adequacy was therefore considered in relation to the precision of the primary agreement estimate and the magnitude of effects detectable with the available sample. With 110 participants, the observed linear-weighted Cohen’s kappa of 0.543 (95% CI: 0.415–0.670) corresponded to an approximate standard error of 0.065 and an estimated power greater than 99% to detect an agreement coefficient of this magnitude against the null hypothesis of κ = 0 at a two-sided α level of 0.05. Because post hoc power calculated from an observed effect provides limited information beyond that conveyed by its confidence interval, the 95% confidence interval was considered the principal measure of statistical precision. Nevertheless, the relatively small numbers of participants within some individual frailty categories limited the ability to detect small subgroup-specific effects. For this reason, the three-category classifications were retained for the primary agreement analysis, whereas the analyses of associated characteristics were dichotomized and considered exploratory.

The analytical sample comprised 110 participants who completed both the EFS and IVCF-20. Seven participants who proceeded to the study assessments but did not complete either the EFS or the IVCF-20 were excluded from the analytical sample, as described in Section 2.1. Among the 110 participants included in the analyses, no missing EFS or IVCF-20 item or total-score data were identified. Analyses of sociodemographic and health-related characteristics were based on the available complete data for the variables required for each analysis.

## 3. Results

### 3.1. Participant Characteristics and Factors Associated with Frailty Classification

A total of 110 community-dwelling older adults were included in the analysis. Most participants were female (74.5%), aged 60–74 years (78.2%), and without a partner (59.1%). Regarding socioeconomic characteristics, 80.0% reported a family income of up to three minimum wages and 62.7% were retired. Most participants rated their health as regular (58.2%), reported no depressive symptoms (77.3%), and had preserved muscle mass (84.5%). Based on the TUG, 39.1% were classified as having low, 51.8% moderate, and 9.1% high risk of falls. According to the FES-I, 16.4% had low, 21.8% moderate, and 61.8% high fear of falling (Table 1).

In the exploratory analyses, the number of children was significantly associated with frailty classification according to both the EFS (*p* = 0.007) and the IVCF-20 (*p* = 0.005). Participants with more than five children represented 25.9% of the EFS frail group and 28.6% of the IVCF-20 frail group, compared with 3.6% and 4.5%, respectively, among participants in the corresponding non-frail groups. Self-perceived health was also associated with classification according to both instruments (EFS, *p* < 0.001; IVCF-20, *p* = 0.016), with poorer self-perceived health occurring proportionally more often among participants classified as frail.

Some associations differed between the instruments. Educational level was associated with EFS classification (*p* = 0.027), but not with IVCF-20 classification (*p* = 0.211). Participants with less than one year of education accounted for 22.2% of the EFS frail group compared with 6.0% of the EFS non-frail group, whereas those with nine or more years of education accounted for 25.9% and 49.4%, respectively. Depressive symptoms were associated with IVCF-20 classification (*p* = 0.021), but not with EFS classification (*p* = 0.075).

No statistically significant associations with either instrument were observed for sex, race/skin color, age group, marital status, family income, retirement status, smoking status, alcohol consumption, muscle mass status, risk of falls, or fear of falling (Table 1). Given the exploratory nature of these analyses and the absence of adjustment for multiple comparisons, these findings should be interpreted cautiously.

### 3.2. Distribution and Cross-Classification of Frailty Categories

The distribution of participants across frailty-related categories differed between the two instruments. According to the EFS, 48 participants (43.6%) were classified as non-frail, 35 (31.8%) as pre-frail, and 27 (24.5%) as frail. According to the IVCF-20, 48 participants (43.6%) were classified as robust, 41 (37.3%) as potentially frail, and 21 (19.1%) as frail (Table 2).

Cross-classification showed that 72 of the 110 participants (65.5%) were assigned to corresponding categories by both instruments. Among the 48 participants classified as non-frail by the EFS, 36 were classified as robust by the IVCF-20. Among the 35 participants classified as pre-frail by the EFS, 21 were classified as potentially frail by the IVCF-20. Of the 27 participants classified as frail by the EFS, 15 were also classified as frail by the IVCF-20. Most discordant classifications occurred between adjacent categories, whereas disagreement between the extreme categories was uncommon (Table 2).

### 3.3. Agreement Between EFS and IVCF-20 Classifications

Exact agreement between the EFS and IVCF-20 classifications was observed in 72 of the 110 participants (65.5%; 95% CI: 56.2–73.7%). When the ordinal nature of the three classification categories was considered, the linear-weighted Cohen’s kappa was 0.543 (95% CI: 0.415–0.670; *p* < 0.001), indicating moderate agreement between the two instruments. The unweighted Cohen’s kappa was 0.464 (*p* < 0.001) (Table 3).

The higher linear-weighted kappa compared with the unweighted kappa is consistent with the cross-classification pattern shown in Table 2, in which most disagreements occurred between adjacent rather than extreme categories.

### 3.4. Correlation Between EFS and IVCF-20 Total Scores

The distributions of the EFS and IVCF-20 total scores departed significantly from normality, as indicated by the Shapiro–Wilk test (EFS: W = 0.966, *p* = 0.007; IVCF-20: W = 0.932, *p* < 0.001). Therefore, Spearman’s rank correlation coefficient was used as the primary measure of association between the total scores of the two instruments.

**Figure 1:**
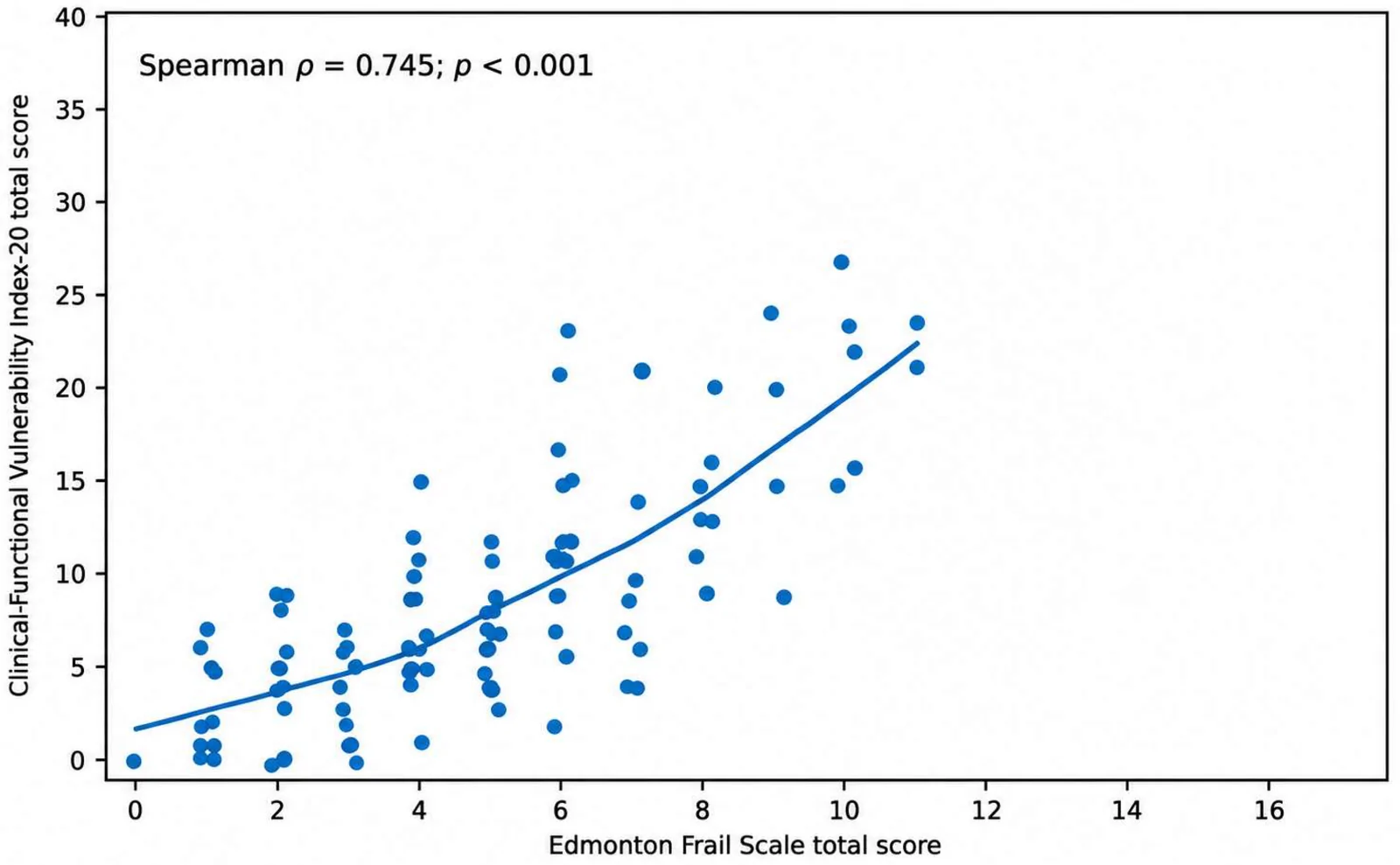
Relationship between Edmonton Frail Scale and Clinical-Functional Vulnerability Index-20 total scores. Each point represents one participant (n = 110). A strong positive monotonic correlation was observed between EFS and IVCF-20 total scores (Spearman’s ρ = 0.745; *p* < 0.001). Slight jittering was applied to the plotted points solely to improve visualization of overlapping observations and did not affect the statistical analysis. The solid line represents a smoothed trend and is included for visualization only. EFS, Edmonton Frail Scale; IVCF-20, Clinical-Functional Vulnerability Index-20.

A strong positive monotonic correlation was observed between EFS and IVCF-20 total scores (Spearman’s ρ = 0.745; *p* < 0.001), indicating that higher EFS scores tended to be associated with higher IVCF-20 scores (Figure 1). As a sensitivity analysis, Pearson’s correlation coefficient yielded a similar result (r = 0.761; *p* < 0.001), supporting the consistency of the observed association.

## 4. Discussion

The present study compared frailty-related classifications obtained with the Edmonton Frail Scale (EFS) and the Clinical-Functional Vulnerability Index-20 (IVCF-20) in community-dwelling older adults. The two instruments showed a strong positive correlation between total scores but only moderate agreement between their three-category classifications. Exact agreement was observed in approximately two-thirds of participants, and most discordant classifications occurred between adjacent categories rather than between the extremes of the classification spectrum. Although the proportion classified in the lowest category was identical for the two instruments, the EFS classified a somewhat larger proportion of participants as frail, whereas the IVCF-20 classified a larger proportion in the intermediate category. Taken together, these findings indicate that the instruments capture related dimensions of vulnerability but do not necessarily assign individual participants to equivalent categories.

The strong correlation observed between EFS and IVCF-20 total scores (Spearman’s ρ = 0.745) indicates that increasing frailty scores on one instrument generally corresponded to increasing vulnerability scores on the other. However, correlation and agreement address different measurement properties. A high correlation reflects the extent to which two measures vary together and does not necessarily imply agreement between the measurements or their resulting classifications [30]. This distinction is particularly relevant in the present study because the strong score correlation coexisted with only moderate categorical agreement (linear-weighted κ = 0.543). The cross-classification matrix provides additional insight into this finding: most discrepancies involved movement between neighboring categories, whereas classifications at opposite extremes were uncommon. Therefore, relying solely on correlation could overstate the degree to which the EFS and IVCF-20 yield interchangeable classifications.

Our findings are broadly consistent with those of Carneiro et al. [14], who directly compared the EFS and IVCF-20 in community-dwelling Brazilian older adults and demonstrated that the instruments identify overlapping but not identical patterns of frailty-related classification. The present study adds to this evidence by explicitly examining the three-category cross-classification and using linear-weighted kappa to account for the ordinal nature of the categories. This approach showed that disagreement was not randomly distributed across the classification spectrum but occurred predominantly between adjacent categories. Such information is clinically relevant because two instruments may produce similar overall distributions while assigning individual participants differently.

Differences in classification are plausible given the distinct structures of the EFS and IVCF-20. Both are multidimensional instruments, but they differ in the domains included, scoring ranges, and classification thresholds [9,11]. The EFS incorporates cognition, general health status, functional independence, social support, medication use, nutrition, mood, continence, and functional performance [9], whereas the IVCF-20 assesses clinical-functional vulnerability through domains including age, self-perceived health, activities of daily living, cognition, mood, mobility, communication, and multiple comorbidities [11]. Consequently, the same participant may accumulate points differently across the two instruments. The higher proportion classified as frail by the EFS (24.5% versus 19.1%) and the higher proportion assigned to the intermediate category by the IVCF-20 (37.3% versus 31.8%) should therefore be interpreted as differences in classification patterns rather than evidence that either instrument is more sensitive, accurate, or conservative. Neither instrument served as a diagnostic reference standard in the present study.

The exploratory analyses identified some participant characteristics associated with the binary frailty classifications. Number of children and self-perceived health were associated with classification according to both instruments. In particular, participants with more than five children represented a greater proportion of the frail groups than of the corresponding non-frail groups. Previous evidence indicates that frailty in community-dwelling older adults is associated with a broad range of demographic, health-related, physical, psychological, and social factors [13,31,32,33]. Nevertheless, the specific association observed with number of children should be interpreted cautiously. The number of participants with more than five children was small, and number of children may reflect several unmeasured social, socioeconomic, cultural, and historical circumstances rather than a direct relationship with frailty. The cross-sectional design also precludes establishing temporal or causal relationships.

Poorer self-perceived health was proportionally more frequent among participants classified as frail by both instruments. Previous research has identified poor self-rated health among the characteristics associated with frailty and with the development or progression of frailty in community-dwelling older adults [13,31]. However, this association requires additional caution in the present comparison because self-perceived health is directly represented within both multidimensional assessment frameworks [9,11]. The observed relationship may therefore reflect, at least in part, conceptual and item-level overlap between the characteristic examined and the frailty-related classifications. Accordingly, these results should not be interpreted as demonstrating that self-perceived health independently predicts frailty.

Some associations differed between instruments. Educational level was associated with the EFS classification but not with the IVCF-20 classification, whereas depressive symptoms were associated with the IVCF-20 classification but not with the EFS. Educational and other socioeconomic characteristics have been associated with frailty in community-dwelling older adults [12,13,31,32,33]. The relationship between depression and frailty is particularly well documented: systematic reviews have identified depressive symptoms as an important factor associated with frailty, and a meta-analysis specifically involving community-dwelling older adults demonstrated a significant relationship between depression and frailty [31,32,33,34]. Nevertheless, the instrument-specific associations observed in the present study should not be interpreted as evidence that one instrument detects socioeconomic or psychological vulnerability better than the other. These exploratory associations should also be interpreted considering potential instrument-content overlap, as both instruments include domains directly or conceptually related to some of the characteristics examined, particularly cognition, mood or depressive symptoms, functional ability and mobility, and self-perceived health. Furthermore, these analyses were exploratory, multiple characteristics were tested without adjustment for multiple comparisons, and the number of participants in some categories was limited. These findings are therefore better viewed as hypotheses for further investigation than as evidence of independent determinants of instrument-specific classification.

Age group, sex, race/skin color, marital status, family income, retirement status, smoking status, alcohol consumption, muscle mass status, TUG-based risk of falls, and FES-I-based fear of falling were not significantly associated with classification according to either instrument in the present sample. This absence of statistically significant associations contrasts with previous evidence linking several demographic, health-related, behavioral, and functional characteristics with frailty in older adults [13,31,32,33]. Fear of falling deserves particular consideration because a systematic review specifically examining community-dwelling older adults found an association between fear of falling and frailty [35]. Thus, the absence of a significant association with FES-I categories in the present study should not be interpreted as evidence that fear of falling is unrelated to frailty. Differences in sample characteristics, frailty instruments, operational definitions, and statistical approaches may contribute to variation across studies. In addition, the present sample was relatively small, predominantly female, and largely composed of younger older adults attending a community center, potentially representing a more functionally active and socially engaged population than older adults recruited in other settings. The binary grouping used for the exploratory analyses, although useful for reducing sparse cell counts, may also have reduced information regarding differences across the original three frailty-related categories.

From a clinical perspective, the findings support viewing the EFS and IVCF-20 as related but distinct multidimensional screening approaches. The moderate weighted agreement indicates meaningful overlap, while the cross-classification results show that classifications should not automatically be treated as interchangeable. This distinction may be relevant when screening results are used to identify individuals who may benefit from further multidimensional assessment, referral, intervention, or longitudinal monitoring. Selection of a screening instrument should therefore consider its intended construct, domains, scoring system, feasibility, and context of application rather than assuming equivalence between instruments [7,8]. In community settings, either instrument may contribute useful information about multidimensional vulnerability, but the present study was not designed to establish the superiority or diagnostic accuracy of one instrument over the other.

Several limitations should be considered when interpreting these findings. First, the cross-sectional design precludes causal inference and does not allow assessment of whether discrepancies between instruments predict different longitudinal outcomes. Second, convenience sampling from a single publicly funded community center in one Brazilian municipality limits generalizability, particularly to homebound or institutionalized older adults, individuals with substantial cognitive or functional impairment, and populations from other geographic and healthcare contexts. Third, the sample size was modest, especially within individual frailty categories, which limited precision for subgroup comparisons and the ability to detect small associations. Fourth, seven individuals who proceeded to the study assessments did not complete either the EFS or the IVCF-20 and were therefore excluded from the analytical sample. Fifth, some variables were self-reported and may be affected by recall or reporting bias. In addition, because several exploratory association tests were conducted without adjustment for multiple comparisons, isolated statistically significant findings should be interpreted cautiously. Finally, both instruments were administered by the same researcher in a fixed sequence, with the EFS preceding the IVCF-20. Although final scores and classifications were calculated only after completion of data collection, formal blinding between instrument administrations was not possible, and potential order or interviewer effects cannot be completely excluded.

Despite these limitations, the study has several strengths. Both instruments were administered to the same participants during the same assessment session and under standardized conditions, allowing a direct head-to-head comparison without differences in sample composition between instruments. The analysis distinguished score correlation from categorical agreement and complemented the weighted kappa with an explicit cross-classification matrix, allowing the location and magnitude of classification discrepancies to be examined. The use of weighted kappa was particularly appropriate because the categories were ordinal and most disagreements occurred between adjacent levels. The study also evaluated the instruments in a real-world community setting, providing evidence relevant to the context in which brief multidimensional screening tools may be used.

Future studies should examine the agreement between the EFS and IVCF-20 in larger and more heterogeneous samples and across different clinical and community settings. Longitudinal designs would be particularly valuable for determining whether discordant classifications are associated with different trajectories of functional decline, hospitalization, disability, or other adverse outcomes. Such studies could clarify whether the instruments provide complementary prognostic information and help determine how differences in classification should be interpreted in clinical practice. Until such evidence is available, the present findings support interpreting EFS and IVCF-20 classifications according to the construct and scoring framework of each instrument rather than treating one as a diagnostic reference standard for the other.

## 5. Conclusions

The EFS and IVCF-20 showed a strong positive correlation between total scores but only moderate agreement between frailty-related classifications among community-dwelling older adults. Although exact agreement was observed in approximately two-thirds of participants, the instruments differed in the distribution of intermediate and frail categories, with most discordant classifications occurring between adjacent categories. These findings indicate that the EFS and IVCF-20 capture related dimensions of frailty and clinical-functional vulnerability but should not be considered interchangeable.

Exploratory analyses showed that number of children and self-perceived health were associated with frailty classification according to both instruments, whereas educational level was associated only with the EFS and depressive symptoms only with the IVCF-20. These associations should be interpreted cautiously given the cross-sectional design, sample size, and multiple exploratory comparisons. Overall, the findings support interpreting each screening instrument according to its specific construct, domains, and classification framework rather than treating either as a diagnostic reference standard for the other. Further studies in larger and more diverse populations, including longitudinal investigations of clinically relevant outcomes and, when validation is intended, comparisons with appropriate reference assessments, are needed to determine the clinical and prognostic relevance of discordant classifications.

## Figures and Tables

**Table 1:** Sociodemographic and clinical characteristics of participants and their association with frailty classification according to the EFS and IVCF-20 (n = 110).

		EFS			IVCF-20		
Characteristics	Total n (%)	Non-Frail n (%)	Frail n (%)	*p*-Value	Non-Frail n (%)	Frail n (%)	*p*-Value
Sex				0.228			0.638
Female	82 (74.5)	59 (71.1)	23 (85.2)		65 (73.0)	17 (81.0)	
Male	28 (25.5)	24 (28.9)	4 (14.8)		24 (27.0)	4 (19.0)	
Race/skin color				0.139			0.054
White	61 (55.5)	49 (59.0)	12 (44.4)		49 (55.1)	12 (57.1)	
Brown	33 (30.0)	25 (30.1)	8 (29.6)		30 (33.7)	3 (14.3)	
Black	16 (14.5)	9 (10.8)	7 (25.9)		10 (11.2)	6 (28.6)	
Age group (years)				0.513			0.062
60–74	86 (78.2)	63 (75.9)	23 (85.2)		69 (77.5)	17 (81.0)	
75–84	21 (19.1)	18 (21.7)	3 (11.1)		19 (21.3)	2 (9.5)	
≥85	3 (2.7)	2 (2.4)	1 (3.7)		1 (1.1)	2 (9.5)	
Marital status				0.251			0.590
With partner	45 (40.9)	37 (44.6)	8 (29.6)		38 (42.7)	7 (33.3)	
Without partner	65 (59.1)	46 (55.4)	19 (70.4)		51 (57.3)	14 (66.7)	
Number of children				0.007			0.005
0	4 (3.6)	3 (3.6)	1 (3.7)		3 (3.4)	1 (4.8)	
1–2	47 (42.7)	39 (47.0)	8 (29.6)		42 (47.2)	5 (23.8)	
3–5	49 (44.5)	38 (45.8)	11 (40.7)		40 (44.9)	9 (42.9)	
>5	10 (9.1)	3 (3.6)	7 (25.9)		4 (4.5)	6 (28.6)	
Education				0.027			0.211
<1 year	11 (10.0)	5 (6.0)	6 (22.2)		7 (7.9)	4 (19.0)	
1–4 years	20 (18.2)	16 (19.3)	4 (14.8)		17 (19.1)	3 (14.3)	
5–8 years	31 (28.2)	21 (25.3)	10 (37.0)		23 (25.8)	8 (38.1)	
≥9 years	48 (43.6)	41 (49.4)	7 (25.9)		42 (47.2)	6 (28.6)	
Family income				0.106			0.141
≤1 MW	46 (41.8)	30 (36.1)	16 (59.3)		34 (38.2)	12 (57.1)	
>1–3 MW	42 (38.2)	35 (42.2)	7 (25.9)		38 (42.7)	4 (19.0)	
>3 MW	22 (20.0)	18 (21.7)	4 (14.8)		17 (19.1)	5 (23.8)	
Retirement status				1.000			0.870
No	41 (37.3)	31 (37.3)	10 (37.0)		34 (38.2)	7 (33.3)	
Yes	69 (62.7)	52 (62.7)	17 (63.0)		55 (61.8)	14 (66.7)	
Self-perceived health				<0.001			0.016
Excellent	14 (12.7)	13 (15.7)	1 (3.7)		13 (14.6)	1 (4.8)	
Good	23 (20.9)	21 (25.3)	2 (7.4)		22 (24.7)	1 (4.8)	
Regular	64 (58.2)	47 (56.6)	17 (63.0)		50 (56.2)	14 (66.7)	
Poor	2 (1.8)	0 (0.0)	2 (7.4)		1 (1.1)	1 (4.8)	
Very poor	7 (6.4)	2 (2.4)	5 (18.5)		3 (3.4)	4 (19.0)	
Smoking status				0.098			1.000
Former	22 (20.0)	13 (15.7)	9 (33.3)		18 (20.2)	4 (19.0)	
Never	78 (70.9)	63 (75.9)	15 (55.6)		63 (70.8)	15 (71.4)	
Current	10 (9.1)	7 (8.4)	3 (11.1)		8 (9.0)	2 (9.5)	
Alcohol consumption				0.234			0.114
Former	5 (4.5)	5 (6.0)	0 (0.0)		5 (5.6)	0 (0.0)	
Never	86 (78.2)	62 (74.7)	24 (88.9)		66 (74.2)	20 (95.2)	
Current	19 (17.3)	16 (19.3)	3 (11.1)		18 (20.2)	1 (4.8)	
Depressive symptoms				0.075			0.021
Absence	85 (77.3)	68 (81.9)	17 (63.0)		73 (82.0)	12 (57.1)	
Presence	25 (22.7)	15 (18.1)	10 (37.0)		16 (18.0)	9 (42.9)	
Muscle mass status				1.000			0.737
Preserved	93 (84.5)	70 (84.3)	23 (85.2)		76 (85.4)	17 (81.0)	
Reduced	17 (15.5)	13 (15.7)	4 (14.8)		13 (14.6)	4 (19.0)	
Risk of falls				0.754			0.634
Low	43 (39.1)	34 (41.0)	9 (33.3)		36 (40.4)	7 (33.3)	
Moderate	57 (51.8)	42 (50.6)	15 (55.6)		46 (51.7)	11 (52.4)	
High	10 (9.1)	7 (8.4)	3 (11.1)		7 (7.9)	3 (14.3)	
Fear of falling				0.573			1.000
Low	18 (16.4)	15 (18.1)	3 (11.1)		15 (16.9)	3 (14.3)	
Moderate	24 (21.8)	19 (22.9)	5 (18.5)		19 (21.3)	5 (23.8)	
High	68 (61.8)	49 (59.0)	19 (70.4)		55 (61.8)	13 (61.9)	

Note: Percentages in the Total column were calculated using the total sample (n = 110) as the denominator; percentages in the EFS and IVCF-20 columns were calculated within each binary classification group. For exploratory association analyses, EFS scores of 0–6 were grouped as non-frail and scores ≥ 7 as frail; IVCF-20 scores of 0–14 were grouped as non-frail and scores ≥ 15 as frail. Pearson’s chi-square test was used when its assumptions were satisfied; Yates’ continuity correction was applied to adequate 2 × 2 tables, Fisher’s exact test to sparse 2 × 2 tables, and Monte Carlo chi-square procedures (200,000 resamples; seed 20260905) to larger sparse contingency tables. No adjustment for multiple comparisons was applied. TUG categories were low (<10 s), moderate (10–20 s), and high (>20 s). FES-I categories were low (16–19), moderate (20–27), and high (28–64). EFS, Edmonton Frail Scale; IVCF-20, Clinical-Functional Vulnerability Index-20; MW, minimum wage; TUG, Timed Up and Go Test; FES-I, Falls Efficacy Scale–International.

**Table 2:** Cross-classification of frailty-related categories according to the Edmonton Frail Scale and the Clinical-Functional Vulnerability Index-20 (n = 110).

	Clinical-Functional Vulnerability Index-20			Total
	Robust	Potentially Frail	Frail	
Edmonton Frail Scale	n (%)	n (%)	n (%)	n (%)
Non-frail	**36 (32.7)**	11 (10.0)	1 (0.9)	48 (43.6)
Pre-Frail	9 (8.2)	**21 (19.1)**	5 (4.5)	35 (31.8)
Frail	3 (2.7)	9 (8.2)	**15 (13.6)**	27 (24.5)
Total	48 (43.6)	41 (37.3)	21 (19.1)	110 (100.0)

Abbreviations: EFS, Edmonton Frail Scale; IVCF-20, Clinical-Functional Vulnerability Index-20. Note: Values are presented as n (%), with percentages calculated using the total study sample (n = 110) as the denominator. EFS categories were defined as non-frail (0–4), pre-frail (5–6), and frail (≥7). IVCF-20 categories were defined as robust (0–6), potentially frail (7–14), and frail (≥15). Bold values on the main diagonal represent participants assigned to corresponding categories by both instruments. Exact agreement was observed in 72 of 110 participants (65.5%).

**Table 3:** Agreement between frailty-related classifications obtained with the Edmonton Frail Scale and the Clinical-Functional Vulnerability Index-20 (n = 110).

Agreement Measure	Estimate	95% CI	*p*-Value
Exact agreement, n (%)	72 (65.5)	56.2–73.7	—
Cohen’s kappa, unweighted	0.464	—	<0.001
Cohen’s kappa, linear weighted	0.543	0.415–0.670	<0.001

Abbreviations: EFS, Edmonton Frail Scale; IVCF-20, Clinical-Functional Vulnerability Index-20; CI, confidence interval. Note: Agreement was assessed across the three ordinal classification categories of each instrument. Linear-weighted Cohen’s kappa was considered the primary agreement coefficient because it accounts for the ordinal nature of the categories and assigns greater weight to disagreements between more distant categories. The unweighted kappa is presented as a secondary analysis. Exact agreement corresponds to participants assigned to corresponding categories by both instruments.

## Data Availability

The datasets analyzed for the current study are not publicly available to protect the participants identities but are available from the corresponding author on reasonable request.

## Abbreviations

The following abbreviations are used in this manuscript:

CI	Confidence Interval
EFS	Edmonton Frailty Scale
FES-I	Falls Efficacy Scale–International
GDS-15	15-item Geriatric Depression Scale
IVCF-20	Clinical-Functional Vulnerability Index-20
MMSE	Mini-Mental State Examination
STROBE	Strengthening the Reporting of Observational Studies in Epidemiology
TUG	Timed Up and Go Test
